# Can performance indicators be used for pedagogic purposes in disaster medicine training?

**DOI:** 10.1186/1757-7241-17-15

**Published:** 2009-03-17

**Authors:** Masahiro Wakasugi, Heléne Nilsson, Johan Hornwall, Tore Vikström, Anders Rüter

**Affiliations:** 1 Centre for Teaching and Research in Disaster Medicine and Traumatology, Faculty of Health Sciences, Department of Clinical and Experimental Medicine, University Hospital, S581 85 Linköping, Sweden; 2 Department of Emergency and Disaster Medicine, Graduated School of Medicine, University of Toyama, 930-0194 Sugitani 2630, Toyama, Japan

## Abstract

**Background:**

Although disaster simulation trainings were widely used to test hospital disaster plans and train medical staff, the teaching performance of the instructors in disaster medicine training has never been evaluated. The aim of this study was to determine whether the performance indicators for measuring educational skill in disaster medicine training could indicate issues that needed improvement.

**Methods:**

The educational skills of 15 groups attending disaster medicine instructor courses were evaluated using 13 measurable performance indicators. The results of each indicator were scored at 0, 1 or 2 according to the teaching performance.

**Results:**

The total summed scores ranged from 17 to 26 with a mean of 22.67. Three indicators: *'Design'*, *'Goal' *and *'Target group' *received the maximum scores. Indicators concerning running exercises had significantly lower scores as compared to others.

**Conclusion:**

Performance indicators could point out the weakness area of instructors' educational skills. Performance indicators can be used effectively for pedagogic purposes.

## Background

Disaster simulation trainings are considered as the traditional method of testing hospital disaster plans and training medical staff, and are widely used throughout the world [1-5]. However, it is still unclear whether these exercises are effective in improving the healthcare provider's skill in disaster response. One reason for this maybe that there is no generally accepted methodology for a quantitative evaluation of these disaster trainings and no scientific evidence of their effectiveness on the healthcare provider's knowledge and skills in disaster response [6].

We have previously introduced and revealed the validity of the performance indicators as a fundamental tool for evaluation and quality control of the staff disaster management skills [7-10]. Measurable performance indicators could be used in training management, command and control at different levels of major incidents and disasters. Well-defined performance indicators assure a fair and unbiased determination of the efficacy of educational methods for disaster medicine training.

Now that we have acquired the tool for testing our educational impact, we have a new question: how do we improve the education methods to achieve more teaching effectiveness? Faculty and staff development has become an increasingly important component of medical education and there is an expanding body of literature to examine the effectiveness of the faculty development course [11,12]. However, to the extent of our knowledge, there are few studies concerning faculty development in disaster medicine [13] and no reports that evaluate the teaching performance of instructors in disaster medicine training. Well-trained instructors are essential for conducting effective disaster medicine training. How can we assess whether instructors have good educational skills? This would be possible if a precise scale, or defined performance indicators for evaluating teaching performance, were established. Thus, the objective of our study was to evaluate whether a postulated set of performance indicators for measuring the teaching skills of instructors in a disaster medicine simulation training course could reveal those parts of education and training that needed improvement.

## Methods

Results from the final examinations of 15 groups participating in a three-day long disaster medicine instructor course were included [14]. The training course was conducted from 2005–2008 by an international training centre and students from 15 different countries registered for it. The training tool used was the Emergo Train System^®^, which is an educational tool consisting of magnetic symbols on white boards; these symbols represent patients, staff and resources, while movable markers are used indicate priority and treatment and a large patient bank with protocol giving the results of treatments based on a trauma score agreed on in Sweden [15].

All students received theoretical and practical training in setting up, running, and evaluating simulation exercises. In the role-play simulation exercises, students were divided into small groups consisting of 2–5 students each and the groups were mixed with regard to the nationalities of the students. One group performed as 'instructors' during an exercise and the other students performed as the target 'students' group. When the exercise was completed, the group members changed roles and trained again. During the role-play exercises, the 'students' groups played the role of average students, not pretending to be extremely bright or poor students. The last of the three exercises in the course was considered as the final exam that we evaluated for this study; the complexity of content and level of difficulty is of this final exercise was higher than those of the first two. The time for setting up the last exercise was three hours, and one hour was allotted for conducting the exercise, including the assessment and feedback.

All the exercises were evaluated according to a template with 13 measurable performance indicators (Table 1). These performance indicators were established as a result of our several years experience conducting instructor training courses. Items were chosen to judge the competencies of trainers in preparing, executing and evaluating skills and knowledge for disaster medicine training. The results were scored as 0, 1 or 2 according to the performance of the 'instructors' group (not scored for individual participants). The maximum possible total score was 26 points for each of the groups. All groups were evaluated by the same persons (the authors of this paper). To avoid inter-rater discrepancies, we standardised the criteria for grading the performance indicators before this study. Throughout the study, one rater was responsible for scoring all the groups of the course. All performances that were evaluated had been previously demonstrated and lectured on to students.

**Table 1:** Proposed performance indicators used in this study, evaluation criteria and points

Indicators	Explanation and comments
1. Design	0 = No clear design
	1 = Clearly described too small or too extensive
	2 = Good

2. Running simulation	0 = No enthusiasm
	1 = Enthusiastic but not with control
	2 = Enthusiastic and in control

3. Aim	0 = Unclear
	1 = Clear but not patient related
	2 = Clear and patient related

4. Goal	0 = Not relevant
	1 = Relevant but not understandable
	2 = Relevant and understandable

5. Objectives	0 = Not stated
	1 = Stated but not measurable
	2 = Stated and measurable

6. Performance indicators	0 = Not realistic
	1 = Realistic but no challenge
	2 = Realistic and challengeable

7. Target level	0 = Not defined
	1 = Defined but not followed
	2 = Defined and followed

8. Target group	0 = Not defined
	1 = Defined but not adopted to
	2 = Defined and adopted to

9. Interventions	0 = No or unclear purpose of interventions
	1 = Clear purpose, poorly executed and/or followed up
	2 = Clear purpose, good executed and followed up

10. Time-out	0 = No start or no stop
	1 = Start and stop no purpose
	2 = Start/Stop/Purpose

11. Evaluation	0 = Not using performance indicators (p.i.)
	1 = Using p.i. Not precise enough
	2 = Using p.i Being specific

12. Feedback	0 = No feed back
	1 = No suggestions on how to improve
	2 = Good feed back, good suggestions

13. Overall impression	0 =
	1 =
	2 =

The statistical method used was Analysis of Variance and the post-hoc Tukey test was used to undertake comparisons in pairs. P < 0.05 was considered as significant.

## Results

All the 13 indicators were evaluated appropriately for the 15 groups. The total summed indicators' scores for each of the simulation exercise ranged from 17 to 26 out of 26 with a mean of 22.67. The median of the summed performance indicators' score was 23. The median values of each evaluated indicator varied from 1.00 to 2.00 out of 2. The value of Cronbach's alpha of the performance indicators was 0.87.

**Figure 1:**
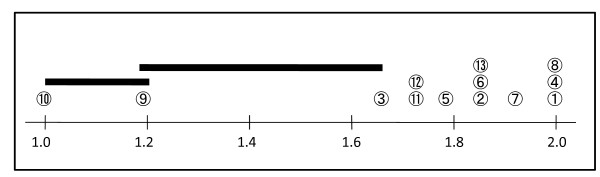
**Comparison of results from 13 different performance indicators**. The mean values of the 13 indicators are on the base line. The numbers of each performance indicators are circled. Numbers that lie below the same horizontal line do not have a significant difference (p < 0.05).

All groups achieved full scores on the three indicators: *1. Design*, *4. Goal *and *8. Target group *(Table 2). The two worst scored indicators (*9. Interventions *and *10. Time out*) significantly differed from the other indicators (Figure 1).

**Table 2:** Average score of each performance indicator of 15 groups

Performance Indicators	Average Score
1. Design	2.00
2. Running simulation	1.87
3. Aim	1.67
4. Goal	2.00
5. Objectives	1.80
6. Performance indicators	1.87
7. Target level	1.93
8. Target group	2.00
9. Interventions	1.20
10. Time outs	1.00
11. Evaluation	1.73
12. Feedback	1.73
13. Overall impression	1.87

Total Average Score	22.67

## Discussion

Although the need to provide training for faculty development to improve the teaching skills of instructors is increasingly recognized in many medical areas [16], their impact has not yet been established. To the extent of our knowledge, there are some studies concerning the usefulness of instructor training in the trauma care education course [17,18]; however, no study has evaluated the impact of the educator's pedagogic skills in disaster medicine. In order to verify the correlation between educator's skill and educational effect for students, it would be necessary to create an objective scale to compare the teaching skill of educators. Thus, for a start, we planned to develop the assessment tools for measuring the educators' teaching skills. We had previously reported the usefulness and effectiveness of performance indicators in evaluating the staff skills during disaster medicine training [7-10]. The same approach could be used to compare the teaching skills quantitatively. Therefore, in this study, we evaluated the educational skills of the participants in the disaster medicine instructor training course by using postulated performance indicators. The indicators used in this study were established based on the results of our experience of the disaster medicine instructor training sessions.

This study elucidated the issues regarding improvements after conducting disaster medicine trainings. The instructor roles for disaster medicine simulation training would be divided into the following three parts. The first part would involve designing the exercise scenario to achieve objectives that were defined clearly and adequately to participants. Next, based on these scenarios, instructors had to conduct the simulation exercise. They introduced the exercise settings and periodically interjected updates, which we referred to as interventions; furthermore, instructors also encouraged participants to discuss focused issues and make decisions within a limited time. Evaluations and feedback were the last task for instructors. They were the key to stimulate the learning process and inform students about their strengths and weak areas that needed improvement. Reviewing results could transform the lessons observed into lessons learned. The performance indicators, as we previously reported, could be used to assess the participants' skills objectively and would assist in giving adequate feedback.

We have chosen performance indicator items in order to be able to evaluate the instructor's skills in the categories of design, execution and evaluation. When we try to apply these categories to the results of this study, fully scored performance indicators would be categorized to the first category that concern preparation for exercises. The designing of the exercise and setting the goal of the adopted exercise to the target group and level were well organized. Although the results fell short of a perfect score, the indicators concerning evaluation and feedback had a relatively favourable grade. Meanwhile, indicators of *Time out *and *Interventions *had significantly worse results than others, as it was more difficult for instructors to conduct and control the simulation exercise properly than other missions such as preparation and evaluation. Training skills requiring expertise in real time interactive methods are less developed than others. To improve the teaching skills of instructors, remediation efforts in this aspect are required. Several possible solutions could be considered for this issue; one is that training the faculty as disaster medicine instructors should be lesson learned, same as the disaster medicine training itself, not lesson observed. Procedural skills are considered to demand a longer practice time than psychomotor skills [19]. Although the techniques and knowledge to design exercises can be obtained from classroom lectures, the skills to conduct and facilitate simulation exercises favourably may need to be learned from substantial experience. These demanding skills may be regarded as general educational skills rather than specific skills for disaster medicine training and need a fair amount of educational experimentation. Further study to compare the results after modification of the faculty development will elucidate this point.

Several limitations of this study should be acknowledged. First, the reliability and validity of the performance indicators need to be considered. Performance indicators in this study were chosen from our experience and lack of strict evidence. Cronbach's alpha, calculated to estimate the reliability, was of an adequately high value to rely on the indicators and we had taken content validity into consideration when choosing the indicator items. However, relationships between the student performance and the education skill of the instructors are our major concern, and future studies to compare these may be needed to validate the performance indicators.

The sensitivity of our performance indicators is the next drawback. Many 'instructors' groups performed well in this study. The majority got a very high score against many of the performance indicators. This may suggest that the postulated performance indicators lack the power to point out the weakness of the instructors' group. Although from another point of view, the reason is that the challenges in this study could have been fairly simple for the 'instructors' groups. This study was conducted as part of a role-play exercise in the instructor training course, different from the usual settings. Participants who acted as 'students' were knowledgeable persons who knew the simulator training system very well. Therefore, we could neither evaluate the primary learning outcome of the trainees nor check the correlation between the instructional skill and the educational impact. The ultimate purpose of the disaster medicine training is to improve patient outcomes as a result of the training program. We are planning another study to elucidate a relation between instructor performance as measured by performance indicators and student performance in a regular disaster training course.

## Conclusion

In conclusion, the performance indicators set in this study could point out the weakness areas of instructors that needed improvement. Future studies may reveal the correlations between the teaching skills of instructors and the educational impact of trainees in disaster medicine training. Performance indicators could be used effectively for pedagogic purposes.

## Competing interests

The authors declare that they have no competing interests.

## Authors' contributions

MW drafted the manuscript, participated in the litterateur search, and in data interpretation. HN and JH participated in data collection and interpretation, TV is head of the Centre for teaching and research in disaster medicine and traumatology, revised the manuscript, and participated in data collection and interpretation. AR conceived of the study and participated in its design and coordination and helped to draft the manuscript. All authors read and approved the final manuscript.
